# Inhibitory Effects and Mechanisms of Luteolin on Proliferation and Migration of Vascular Smooth Muscle Cells

**DOI:** 10.3390/nu5051648

**Published:** 2013-05-17

**Authors:** Dehua Jiang, Dongye Li, Wanling Wu

**Affiliations:** Research Institute of Cardiovascular Diseases, Xuzhou Medical College, Xuzhou 221002, China; E-Mails: yiyuanerzhu1021@163.com (D.J.); wanlinggrose@163.com (W.W.)

**Keywords:** atherosclerosis, luteolin, migration, proliferation, vascular smooth muscle cells (VSMCs)

## Abstract

Atherosclerosis (AS) is a complicated progress, involving many types of cells. Although the exact mechanisms of progression of atherosclerosis are uncertain, the balance of vascular smooth muscle cells (VSMCs) proliferation and apoptosis appears to play a pivotal role in the pathogenesis and progression of atherosclerosis, and much discussion has been undertaken to elucidate the detailed mechanisms, relevant gene expression and transduction pathways. Drug treatment has focused on ameliorating atherosclerosis. Some researchers have indicated that inhibiting VSMCs proliferation is involved in attenuating atherosclerosis. Luteolin is a kind of flavonoids naturally occurring in many plants and possesses beneficial effects on cardiovascular diseases. Luteolin can reduce VSMCs’ proliferation and migration and this reduction is stimulated by several factors. The aim of this review is to summarize the existing inhibitory effects and mechanisms of luteolin on proliferation and migration of VSMCs, and consider whether luteolin may be a potential candidate for preventing and treating atherosclerosis.

## 1. Introduction

The abnormal proliferation and migration of vascular smooth muscle cells (VSMCs) have been implicated to play a key role in a number of cardiovascular pathologies, such as in the pathogenesis of hypertension, the development of restenosis after percutaneous coronary intervention (PCI) and the progression of atherosclerosis [1,2]. As one of the leading causes of death and disability in industrialized society, atherosclerosis is a multi stage and multi-factorial disease involving genetic, environmental, psychosocial and other factors. Some features of atherosclerosis remain to be studied. Atherosclerosis has been thought to be initiated by inflammatory mechanisms. However, in early as well as in advanced lesions, vascular proliferation plays a pivotal role in disease progression. Data has suggested that some characteristics of atherosclerosis are similar to cancer [3]. In accordance with these findings, the “monoclonal” hypothesis of atherosclerosis has been proposed. Therefore, the current knowledge of the pathogenesis mechanisms of atherosclerosis is complicated.

Endothelial dysfunction, inflammation, cell proliferation, and vascular remodeling characterize atherosclerosis followed by early plaque development, plaque rupture, thrombosis of the affected vessel, and infarction of tissues and organs [4]. The normal arterial endothelium plays a crucial role in maintaining normal vessel wall function by having the capability to inhibit thrombus formation, leukocyte adhesion, and VSMCs proliferation, and by regulation of vascular tone. Endothelial cell damage stimulates the formation of the atherosclerotic plaque and vessel wall inflammation, which are major contributors to the etiology of atherosclerosis [5]. As part of the innate immune system, endothelial cells respond to several triggers by producing cell surface adhesion molecules, chemokines and inflammatory cytokines. These characteristics of endothelial cell dysfunction form an initial step in atherosclerosis development. When endothelial cells are injured, platelets aggregate and adhere to the injured cell surface and initiate the thrombosis process, which participates in the development and progression of atherosclerosis. Aggregated platelets could release platelet-derived growth factor (PDGF) which stimulates VSMCs migration and proliferation and in turn stimulates the initiation and proliferation of atherosclerosis. As one of the major producers of the extracellular matrix within the vessel wall, VSMCs also produce many cytokines such as PDGF, transforming growth factor-β and so on, all of which contribute to the initiation and propagation of the inflammatory response to lipid. Previous studies already show that exaggerated and uncontrolled VSMCs proliferation appears to be a feature of atherosclerosis. In recent years, other studies found that apoptosis of VSMCs has also played an important role in restenosis after PCI. Although the role is controversial, most scholars generally agree that there is excessive proliferation of VSMCs when apoptosis is inadequate in atherosclerosis [6]. Imbalance between VSMCs’ proliferation and apoptosis accelerates the development of atherosclerosis. It is well known that proliferation of VSMCs and vascular smooth muscle foam cells within the lesions are apparent, resulting in vessel occlusion. Progression of atherosclerosis is characterized by atheroma instability and plaque disruption, followed by local thrombosis, which constitutes the clinical indications of acute coronary syndrome [7].

Until now, emerging evidence has shown remarkable progress in tackling atherosclerosis. Some of the drugs, such as 3-hydroxy-3-methylglutaryl-coenzyme A (HMG-CoA) reductase inhibitors and calcium channel blockers, have already tested positive in inhibiting atherosclerosis [8]. The discovery of these major anti-atherosclerotic mechanisms has prompted the exploration of new drugs to protect against atherosclerosis. 

In recent years, the use of herbal remedies to prevent and treat atherosclerosis has received much attention [9]. Dietary polyphenolic compounds such as flavonoids are important candidates for chemopreventive agents [10]. However, only a few studies have evaluated and demonstrated the anti-atherosclerotic effect of flavonoids. In human studies, wide variability has been observed in the effects of flavonoids on these biomarkers of cardiovascular disease risk. This heterogeneity reflects variation in the bioavailability of flavonoid subclasses [11]. Many previous *in vitro* studies examining the bioactivity of flavonoids have failed to consider the effects of metabolic transformation on flavonoid activity. Loke *et al.* examined the effect of quercetin and its major metabolites on the production of pro-inflammatory eicosanoids by human leukocytes [12]. They found that structural modification of quercetin due to metabolic transformation had a profound effect on bioactivity. The structural features required for antioxidant activity of quercetin and related flavonoids were unrelated to that required for inhibition of inflammatory eicosanoids. That is to say, the results for quercetin showed a lack of association between antioxidant and lipoxygenase inhibitory activity. Meanwhile, Chen *et al.* elucidated the mechanisms that caused the pharmacokinetic difference between luteolin and apigenin in rats [13]. They found that luteolin and apigenin are very similar in structure; however, one-hydroxyl difference gives those different characteristics in absorption and metabolism, which results in a much lower exposure of luteolin than apigenin when Flos Chrysanthemi extract (FCE) is orally administered to rats. The authors believe that flavonoids could behave as antioxidants or pro-oxidants, depending on the concentration and the source of the free radicals [14]. Luteolin is a natural anti-oxidant with less pro-oxidant potential than the flavonol quercetin, the best-studied flavonoid, but apparently with a better safety profile. Moreover, when compared to other flavonoids, luteolin was usually among the most effective in terms of antioxidant aspects [15]. In an earlier evaluation of the same study population after the 10-year follow-up, Yochum *et al.* had analyzed single flavonols and flavones [16]. Quercetin and kaempferol significantly reduced coronary heart disease-induced death, while luteolin was at the border of significance [OR, 0.8 (0.63–1.02)]. Plants rich in luteolin have used for the prevention and treatment of various diseases, such as hypertension, infective diseases and cancer [17,18]. In addition, many experimental studies have shown that luteolin has a series of biological and pharmacological activities, including antioxidants, anti-inflammatory, antibacterial and anticancer activity [19], which are functionally associated. For instance, the anti-inflammatory activity may link to its anti-apoptotic property. Whether luteolin is a potentially useful drug in treating atherosclerosis relevant to VSMCs disorders has not been well documented [20,21]. Some effects of luteolin have been well documented in various cell types, such as cancer cells and heart cells and reviewed for many diseases, especially in cancers, but no reviews exist describing the direct inhibitive effects and mechanisms of luteolin on proliferation and migration in VSMCs. Thus, the subject area is of interest. This review explains that luteolin can be regarded as an agent for treating atherosclerosis partly through its anti-migration and anti-proliferative effects on VSMCs.

## 2. Luteolin and Atherosclerosis

Despite impressive gains in diagnosis and treatment, atherosclerosis remains a serious clinical issue and threat to public health. Luteolin is one of the most common flavonoids present in edible plants and in plants used in traditional medicine to treat a wide variety of pathologies. A member of the flavones group of flavonoids, luteolin consists of two benzene rings, a third, oxygen-containing ring, and a 2–3 carbon double bond, it also consists of hydroxyl groups at carbons 5, 7, 3′, and 4′ positions [22]. A structure-activity study indicated that the position, number and substitution of the hydroxyl group of the benzene rings, and saturation of the C2–C3 bond are important factors affecting flavonoid inhibition of phosphatidylinositol 3′-kinase (PI3K) [23]. Luteolin exhibits versatile biological effects in animal studies. This is likely to be attributable to the fact that luteolin is a molecule with many targets. Luteolin possesses diverse health benefits in cases of allergy, cancer, and respiratory diseases. One of the most well known benefits of luteolin is for cardiovascular health [24]. Luteolin may have the capacity to interact with multiple molecular targets, which involve diverse intracellular pathways. To investigate whether luteolin has preventative effects on atherosclerosis relevant to VSMCs disorders and other cardiovascular diseases, we and other researchers studied the role and mechanism of luteolin in several cell types, such as cardiomyocytes [25], VSMCs [26,27], endothelial cells [28] and macrophages [29]. Cardiomyocyte ischemic damage in rat has been prevented by pretreatment with luteolin [30]. This involved up-regulation of Akt phosphorylation, which caused the ratio of Bcl-2/Bax to increased. Other ways by which luteolin could exert its anti-apoptotic and cardio-protective effects include raised fibroblast growth factor receptor 2(FGFR2) and leukemia inhibitory factor(LIF) expression and increased BAD phosphorylation [31]. In addition, luteolin has been shown to inhibit apoptosis and improvs cardiomyocyte contractile function at least partly through the PI3K/Akt pathway in simulated ischemia-reperfusion (I/R) [32]. Luteolin also possesses a vasodilator effect due to the increases in the expression in human vascular endothelial cells of endothelial nitric oxide synthase, an enzyme responsible for synthesizing the potent vasodilator nitric oxide. Meanwhile, a recent report demonstrated that luteolin inhibits apoptosis in endothelial cells induced by lysophosphatidylcholine (LPC) [33] and angiotensin II (Ang II) [28]. These reports suggested that luteolin might have a potential effect against atherosclerosis.

## 3. Luteolin Inhibited VSMCs Proliferation and Migration

### 3.1. Luteolin Inhibited VSMCs Proliferation

Previous studies had shown that luteolin could inhibit VSMCs’ proliferation and migration [26,27]. Diverse signal transduction systems have been proposed to translate the mitogenic stimulus within VSMCs that control VSMCs’ proliferation; among them the mitogen activated protein kinase (MAPKs) [34], nuclear factor kappa (NFκ)-B, protein kinase G (PKG) or the PI3Kpathways [35]. For example, the Akt signaling pathway has been considered as one of the most important pathways involved in regulating cell survival. It is involved in angiotensin II and PDGF induced hypertrophy and migration of VSMCs. Akt is essential for VSMCs’ proliferation and migration, and the ablation of Akt leads to a severe lesion in atherosclerosis and occlusive artery disease [36]. 

To examine the latent mechanism of the anti-proliferative effect exerted by luteolin, Kim *et al.* tested the effects of luteolin on rat VSMCs in culture and aimed to explore the mechanisms by which the cell cycle was affected by luteolin [26]. In *in vitro* experiment in rats, they measured cell cycle phase and some involved signaling transduction pathways in primary rat aortic VSMCs, which stimulated by PDGF. The experiment showed that luteolin inhibited PDGF-BB-induced proliferation and DNA synthesis of VSMCs in a concentration-dependent behavior. In addition, flow cytometry analysis of DNA content indicated that luteolin reduced the PDGF-BB inducible cell cycle progression. PDGF-treated cells were associated with a significant increase in the number of cells in the G_2_/M phase and S phase, as well as a decrease in the number of cells in the G_0_/G_1_ phase. In contrast, after incubation with luteolin for 24 h, the percentage of cells in S phase decreased. Western blot analysis showed that luteolin significantly inhibited ERK1/2 phosphorylation induced by PDGF-BB in a concentration-dependent manner. Similarly, pretreatment with luteolin also inhibited Akt phosphorylation and PLC-γ phosphorylation induced by PDGF-BB in a concentration-dependent manner. In addition, U0126, LY294002 and U73122 were used as the positive controls, and significantly inhibited ERK1/2, Akt and PLC-γ phosphorylation, respectively. Meanwhile, pretreatment with luteolin significantly inhibited PDGF-Rβ tyrosine phosphorylation induced by PDGF-BB in a concentration-dependent manner. Moreover, reverse transcription polymerase chain reaction (RT-PCR) analysis showed the c-fos mRNA expression down regulated by luteolin in a concentration-dependent manner. Consistent with these results, Kim *et al.* concluded that the inhibitory effect of luteolin on the PDGF-BB-induced proliferation of rat aortic VSMCs may be mediated by blocking phosphorylation of PDGF receptor beta (PDGF-Rβ) [26].

We have investigated the anti-proliferative effect of luteolin and its potential mechanism on VSMCs stimulated by hydrogen peroxide (H_2_O_2_) [27]. We also observed VSMCs proliferation and cell viability reduced on pre-incubation with luteolin using MTT method or by cell counting analysis. To demonstrate whether luteolin inhibited H_2_O_2_-induced VSMCs proliferation by involving the activation of Akt signals, we analyze the phosphorylation levels of PDK1, p-Akt (308) and p-Akt (473) in response to H_2_O_2_ after pre-treatment with luteolin for 12 h. Western blot analysis showed that luteolin down regulated the expression levels of PDK1, p-Akt (308) and p-Akt (473). Src kinase signaling is involved in the atherogenic responses in VSMCs. Similarly, H_2_O_2_-stimulated Src phosphorylation has suppressed by luteolin. This study proved that the proliferation of VSMCs could be reduced by luteolin. We concluded that luteolin’s inhibition of H_2_O_2_-induced VSMCs’ proliferation is partially due to suppressing the Src and Akt signaling pathways. 

The VSMCs’ excessive proliferation and intimal migration is an important pathological factor leading to atherosclerosis [37,38]. Particular MAPKs have different and often contradictory effects in the control of the proliferation/apoptosis ratio. Perez-Vizcaino *et al.* compared the effects of quercetin in intimal- and medial-type rat VSMCs in culture [39]. They observed the reduced viability of a polyclonal intimal-type cell line derived from neonatal aorta pre-incubation with quercetin but not of a medial-type cell line derived from adult aorta. Quercetin also preferentially reduced the viability of intimal-type over medial-type VSMCs in primary cultures derived from balloon injured carotid arteries. They compared the expression of MAPKs (ERK, p38, and JNK) and its phosphorylation between intimal- and medial-type VSMCs. The expression of p38 and JNK are similar in the two cell types. Medial-type VSMCs showed a weak JNK phosphorylation while this markedly increased in intimal-type VSMCs. They concluded that quercetin preferentially produced apoptosis in intimal-type compared to medial-type VSMCs. However, a comparative study should be made to determine whether luteolin possesses similar effects in intimal- and medial-type rat VSMCs. 

Cell-cycle control is a highly regulated process that involves a complex cascade of cellular events including activation of cyclins and cyclin-dependent kinase inhibitors (CDKs). In mammalian cells, cell growth is governed by cell cycle, which is a complex and stepwise progression where a growth signal turns on an interaction between CDKs and cyclins, activating the CDKs, which phosphorylate Rb to release E2F transcription factors, in order to regulate the expression of various genes required for G_1_-S phase transition. Luteolin is able to arrest the cell cycle during the G_1_ phase in human gastric and prostate cancer, and in melanoma cells [40]. The G_1_ cell cycle arrest induced by luteolin is associated with inhibition of the CDK2 activity in melanoma OCM-1 and colorectal cancer HT-29 cells [41]. Similarly, as the cell cycle is a final common pathway in VSMCs’ proliferation, proteins of the cell cycle have emerged as logical targets for the treatment and prevention of atherosclerosis [42,43,44,45]. The CDKs such as rapamycin can induce G_1_ arrest in cultured VSMCs, and inhibit intimal hyperplasia. However, the role of luteolin on cell-cycle control in VSMCs has been a neglected area.

### 3.2. Luteolin Inhibited VSMCs Migration

After migrating to the intima, VSMCs lose their original function of controlling the contraction of blood vessels and accumulate in the inner vessel; this ultimately exacerbates atherosclerosis [46]. As an important and well-recognized process in the development of atherosclerosis, VSMCs migration may be affected at a number of steps. One promising area of research appears to be the controlling of signal transduction processes. We have investigated whether luteolin possesses the anti-migration effect of luteolin on VSMCs stimulated by H_2_O_2_. Transwell migration analysis measured the effect of luteolin on the migration of VSMCs treated with H_2_O_2_. The migration of VSMCs from the upper to the lower chamber was visibly promoted with H_2_O_2_ [27]. In this study, the migration promoting effect of H_2_O_2_ was significantly abolished by pre-treatment with luteolin. Lamy *et al.* presented evidence that apigenin and luteolin act as potent inhibitors of PDGFR-β activities, leading to an inhibition of PDGF-BB induced pulmonary artery smooth muscle cells migration and invasion [47]. Inhibition of PDGFR-β activity by apigenin and luteolin occurred at low concentrations of the molecules and resulted in the inhibition of ERK and Akt phosphorylation triggered by PDGF, as well as in a marked reduction of the migratory and invasive properties of these cells.

### 3.3. Luteolin and Apoptosis of VSMCs

Cell death is linked with the pathophysiology of cardiovascular diseases, including myocardial hypertrophy, atherosclerosis and heart failure. Consistent with proliferation and migration of VSMCs, apoptosis of VSMCs is also important to the pathogenesis of atherosclerosis [48]. Although these association studies are interesting, it has been very difficult to study the direct consequences of VSMCs apoptosis *in vivo* because of the lack of a model in which VSMCs-specific apoptosis is induced within the vessel wall [49]. Moreover, it is not clear what is the significance of the relatively low frequencies of VSMCs apoptosis in human atherosclerosis. It is obvious that distinct mechanisms for modulating cellular signaling pathways exist in different cells. For example, luteolin activates Akt in cardiac cells [29,30,31], whereas it suppresses Akt in VSMCs [26,27]. Horinaka *et al.* observed luteolin-induced apoptosis in malignant cells but not in normal human peripheral blood mononuclear cells [50]. That is to say, luteolin possesses distinct mechanisms for modulating cellular signaling pathways that exist in normal cells and in malignant cancer cells. For cancer cells, luteolin-induced apoptosis is associated with luteolin’s ability to induce activation of wild-type p53, to imbalance the Bcl-2 family of proteins, to up-regulate death receptor 5 and to promote STAT3 degradation [51]. Unlike cancer cells, there is a lack of direct evidence supporting the connection between the anti-atherogenic effect of luteolin and apoptosis signaling pathways, such as Bcl-2, Bax, Caspase and its role in VSMCs. 

**Figure 1:**
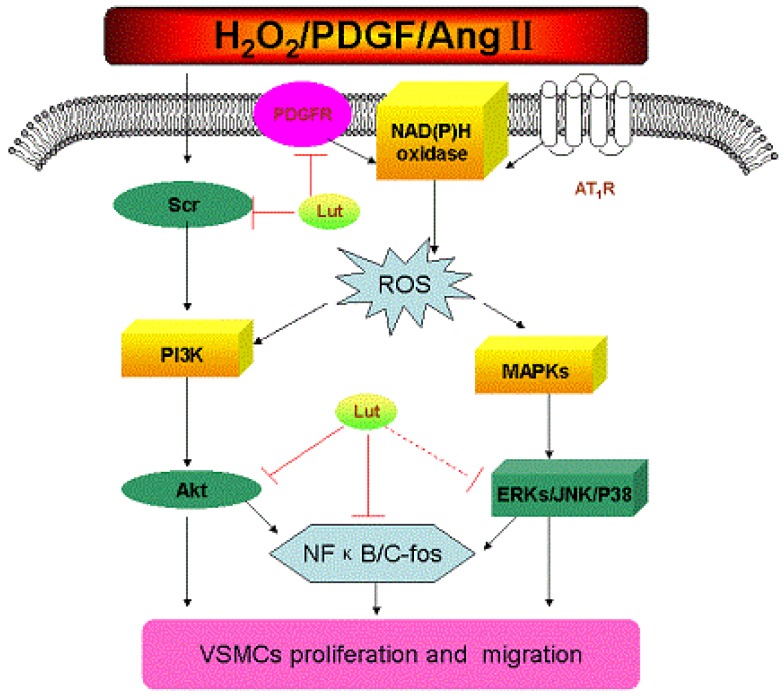
Possible mechanisms of luteolin inhibit vascular smooth muscle cells’ proliferation and migration. (AT 1R: angiotensin type 1 receptor; Lut: Luteolin).

Consistent with these results, these findings suggest luteolin may prove to be a potential therapeutic agent for the prevention and possible treatment of atherosclerosis relevant to VSMC disorders (Figure 1).

Black arrows indicate the main signaling pathways about VSMCs’ proliferation and migration in response to several stimulations such as H_2_O_2_, PDGF-BB and Ang II. In response to this stimulation, ROS can be produced by VSMCs and stimulate MAPKs and Akt. This picture shows that luteolin inhibited H_2_O_2_-induced VSMCs’ proliferation partially due to suppressing the Src and Akt signaling pathways. Meanwhile, luteolin significantly inhibited the PDGF-BB induced ERK1/2, Akt activation as well as c-fos gene expression. Moreover, luteolin can block phosphorylation of PDGF-Rβ.

## 4. Conclusions and Perspective

The persistently high morbidity and mortality rates of cardiovascular diseases such as atherosclerosis and restenosis indicate that the current understanding of the molecular mechanisms responsible for the pathogenesis of atherosclerosis is incomplete. The VSMCs is a major cellular component of the blood vessel wall, and its primary physiological functions are to maintain homeostasis of blood flow and blood pressure within normal ranges. Several experimental evidences indicate that luteolin can protect against different diseases. The protective role of luteolin is supported by detailed findings at the cellular and molecular levels. Due to the non-selective nature of luteolin, more basic research and clinical studies are necessary in order to ensure the safety and efficacy of luteolin and for ascertaining the optimum doses for prevention and treatment. In addition, Luteolin exhibits versatile biological effects. Luteolin inhibits platelet function through binding to the thromboxane A2 receptor [52] and inhibits vascular endothelial growth factor-induced angiogenesis by targeting PI3K activity [53]. Oral administration of luteolin caused a significant increase in uterine weight in immature ovariectomised rats. It also caused a significant increase in uterine diameter, thickness of the endometrium and its epithelial cell height when compared with those of control rats. However, along with ethinyl estradiol, they exhibited slight anti-estrogenic activity [54]. Xu *et al.* reported that luteolin possess a vascular relaxation effect in porcine coronary artery by Structure–activity relationships analysis [55]. It revealed that for good relaxation activity, the 5-OH, 7-OH, 4-OH, C2=C3 and C4=O functionalities were essential. How to modify the structure of luteolin to improve the selectivity and potency of this compound needs further study. Though many works need to be done, the research on luteolin may be interesting to pharmaceutical intervention in atherosclerosis. 

This review was limited in several respects. Firstly, there is a lack of direct evidence supporting the connection between anti-atherogenic effect of luteolin and apoptosis signaling pathways in VSMCs. That is to say, the significance of this potential connection is unclear, and the mechanisms by which luteolin regulates the balance between VSMCs’ proliferation and apoptosis has not been elucidated. Secondly, the initiation and early progression of atherosclerosis attribute largely to interactions among macrophages, lymphocytes, VSMCs and endothelial cells. Endothelial cell injury is the fundamental stimulus responsible for the formation of the atherosclerosis. Proliferation and migration of VSMCs in the intima is a major contributor to the processing of atherosclerosis. Meanwhile, macrophage infiltration is also involved in the initiation and progression of atherosclerosis. How to predict the interaction effects of luteolin among various cells involving atherosclerosis is not clear. In addition, studies could be expected that investigate how to explain the role of luteolin and whether luteolin has potential side effects such as hypotension, bleeding, tissue ischemia, gender differences for contradiction in pregnant women and in cardiac treatment. Therefore, it may still be premature to draw any conclusion regarding its implication. In conclusion, although some anti-atherogenic mechanisms are known for luteolin, all mechanisms mediated by luteolin must be known to understand the true value of this compound as a potential therapeutic agent for atherosclerosis.
